# Sacrificed Lives

**Published:** 1869-01

**Authors:** 


### SACRIFICED LIVES.

If a baby pokes its tiny fingers into a burning candle once, only once, it will never do it again.

If you take a dog into a house and tie a tin pan to his tail, you will not soon, if ever, get him to enter that house again, voluntarily.

There is reason to believe, that man is the only animal requiring repeated experience in his own case, and a multitude of experiences of others, to learn valuable practical lessons for the every day conduct of life. Many a person has been shot dead on the spot, by having a gun or pistol pointed at him and the trigger pulled, the person doing it thinking it was not loaded, and yet new occurrences of the kind are frequently taking place; the rule should be, never to point a gun towards a living person, even though it has neither trigger nor barrel; then you may be sure that you will never shoot a friend.

### DEATH BY RAIL.

Scarce a day passes in which a person is not maimed for life, or killed outright, by leaving a rail-car while it is in motion. Within a week, a gentleman of wealth, who had been in a place of high public trust for twenty years, jumped from a car which was running faster than he supposed; he was jerked around under the wheels, both legs were taken off, and his mutilated dead body was carried into his own house, near by, which he had left a few hours before in good health. No pen can describe the agony of that bereaved wife, of those fatherless sons and daughters. The efficient remedy is, if you can't possibly wait two minutes or so longer, to allow the car to come to a full stop, at least take the precaution to step out from the rear platform, and make this an *invariable* rule.

### HE WOULD DO IT.

Going along Fourth Avenue the other day, we saw a cart on which was a man's leg; it was still bleeding, and had not been cut off properly, or rather, scientifically. Looking a little farther for the remainder of the body, we saw that it was all smashed into a jelly. The man had been run over by a large car. He had attempted to cross the street, immediately in front of a passing vehicle; in his hurry he stumbled, and staggering forward fell immediately across the track of a rail-car which happened to be passing at the time. There is scarcely a child in New York which has not been taught the danger of crossing the street in front of a moving horse or vehicle. This man knew the danger, and being a New Yorker, had read of many fatal occurrences of this kind, and because he had not been killed himself in that way—"he would do it."

